# Matrine Impairs Platelet Function and Thrombosis and Inhibits ROS Production

**DOI:** 10.3389/fphar.2021.717725

**Published:** 2021-07-22

**Authors:** Sixuan Zhang, Xiang Gui, Yangyang Ding, Huan Tong, Wen Ju, Yue Li, Zhenyu Li, Lingyu Zeng, Kailin Xu, Jianlin Qiao

**Affiliations:** ^1^ Blood Diseases Institute, Xuzhou Medical University, Xuzhou, China; ^2^ Department of Hematology, The Affiliated Hospital of Xuzhou Medical University, Xuzhou, China; ^3^ Key Laboratory of Bone Marrow Stem Cell, Xuzhou, China; ^4^ School of Medical Technology, Xuzhou Medical University, Xuzhou, China

**Keywords:** matrine, platelet, thrombosis, ROS, hemostasis

## Abstract

Matrine is a naturally occurring alkaloid and possesses a wide range of pharmacological properties, such as anti-cancer, anti-oxidant, anti-inflammatory effects. However, whether it affects platelet function and thrombosis remains unclear. This study aims to evaluate the effect of matrine on platelet function and thrombus formation. Human platelets were treated with matrine (0–1 mg/ml) for 1 h at 37°C followed by measuring platelet aggregation, granule secretion, receptor expression by flow cytometry, spreading and clot retraction. In addition, matrine (10 mg/kg) was injected intraperitoneally into mice to measure tail bleeding time, arterial and venous thrombus formation. Matrine dose-dependently inhibited platelet aggregation and ATP release in response to either collagen-related peptide (Collagen-related peptide, 0.1 μg/ml) or thrombin (0.04 U/mL) stimulation without altering the expression of P-selectin, glycoprotein Ibα, GPVI, or αIIbβ3. In addition, matrine-treated platelets presented significantly decreased spreading on fibrinogen or collagen and clot retraction along with reduced phosphorylation of c-Src. Moreover, matrine administration significantly impaired the *in vivo* hemostatic function of platelets, arterial and venous thrombus formation. Furthermore, in platelets stimulated with CRP or thrombin, matrine significantly reduced Reactive oxygen species generation, inhibited the phosphorylation level of ERK1/2 (Thr202/Tyr204), p38 (Thr180/Tyr182) and AKT (Thr308/Ser473) as well as increased VASP phosphorylation (Ser239) and intracellular cGMP level. In conclusion, matrine inhibits platelet function, arterial and venous thrombosis, possibly involving inhibition of ROS generation, suggesting that matrine might be used as an antiplatelet agent for treating thrombotic or cardiovascular diseases.

## Introduction

Platelets play key roles in pathological thrombosis and physiological hemostasis. In response to vascular injury, platelets will attach to the sub-endothelial matrix via recognition of exposed collagen and von Willebrand factor (VWF) by platelet surface adhesive receptors, glycoprotein (GP) Ib-IX-V and GPVI (Qiao et al., 2010; Qiao et al., 2018a). Ligands binding to these platelet receptors will initiate intra-platelet signaling pathway transduction, resulting in the activation of integrin α_IIb_β_3_ (inside-out signaling), which binds fibrinogen, fibronectin or VWF and mediates platelet aggregation (Li et al., 2010; Bye et al., 2016). Meanwhile, binding of α_IIb_β_3_ to ligands also trigger a series of intra-platelet signaling events (outside-in signaling), leading to the tyrosine phosphorylation of several signaling proteins, including c-Src, spleen tyrosine kinase (Syk), phospholipase Cγ2 (PLCγ2), which modulates granule secretion, platelet spreading, clot retraction and stabilization of thrombus formation (Shattil et al., 1998; Durrant et al., 2017). Considering the critical roles in hemostasis, impaired platelet function or abnormal expression of platelet surface receptors might contribute to platelet disorders such as bleeding (Scharf, 2003; Bolton-Maggs et al., 2006).

As a Traditional Chinese Medicine (TCM), *Kushen* is the dry root of the leguminous plant *Sophora flavescens* Aiton (He et al., 2015) and has been commonly used for the treatment of several diseases as a TCM with a long history in China, such as tumors (Sun et al., 2012). At present, the compound *Kushen* injection has been used as an adjuvant therapy for the treatment of multiple cancers in clinic (Ma et al., 2016; Wang et al., 2016; Yang et al., 2018; Ao et al., 2019). As the main bioactive compound in *Kushen*, matrine is a naturally occurring alkaloid and has been demonstrated to possess a wide range of pharmacological effects, such as anti-cancer, anti-oxidant, anti-inflammatory, anti-bacterial, anti-virus, and anti-fibrotic properties (You et al., 2020; Zhang et al., 2020). Due to these pharmacological properties, the role and effect of matrine has been investigated in several diseases, such as cardiovascular diseases (Yu et al., 2014; Zhang et al., 2021), liver diseases (Gao et al., 2018), autoimmune diseases (Zhao et al., 2011; Kan et al., 2015), or multiple cancers (Liu et al., 2014a). Several studies have shown that the main mechanism by how matrine exerts anti-cancer activity is through inhibiting cancer cell proliferation and metastasis, inducing cancer cell apoptosis, reversing the drug resistance and reducing toxicity of anticancer drugs (Zhang et al., 2020). Apart from anticancer activity, matrine also possesses activities in other systems, such as nervous system, immune system and cardiovascular system, with the possible mechanisms being through inhibition of inflammation, reduction of oxidative stress-mediated damage or regulation of autophagy or apoptosis (Zhang et al., 2020). Considering the broad spectrum activities, whether matrine affects platelet function and thrombus formation remains poorly understood.

In the present study, we aim to investigate the effect of matrine on platelet activation and function through incubation of isolated platelets with different concentrations of matrine. In addition, the effect of matrine on *in vivo* hemostatic function of platelets and thrombus formation was also assessed.

## Materials and Methods

### Reagents

Matrine was purchased from MedChemExpress (Monmouth Junction, NJ, United States) with a purity ≥98% and dissolved in saline. Collagen-related peptide (CRP) was prepared as previously described (Arthur et al., 2012). Collagen and thrombin (≥10 NIH units/vial) were from Chrono-log Corporation (Havertown, PA, United States). Fibrinogen and BSA (bovine serum albumin) were purchased from Sigma-Aldrich (St. Louis, MO, United States). Mepacrine (also known as Quinacrine) was from APExBIO (Boston, MA, United States). FITC-conjugated mouse anti-human CD41a and PAC-1 antibody were from BD Biosciences (San Jose, CA, United States). PE-labelled anti-human/mouse CD62p (P-Selectin) and anti-human Glycoprotein VI antibody were purchased from eBioscience (San Diego, CA, United States). Alexa Fluor-546-labelled phalloidin was purchased from Thermo Fisher Scientific (Waltham, MA, United States). FITC-conjugated goat anti-mouse IgG was purchased from ZSGB-BIO (Beijing, China). β-actin antibody and HRP-conjugated anti-rabbit IgG were purchased from Cell Signaling Technology (Danvers, MA, United States).

### Animals

All experimental procedures involving animals were complied with ARRIVE guidelines and approved by the Ethic Committee of Xuzhou Medical University. C57BL/6 mice with an age of 8–10 weeks and weight of 24–28 g were purchased from SLAC Laboratory Animal Co., Ltd. (Shanghai, China). All mice were housed in specific pathogen free (SPF) grade environment with free access to food and water.

### Platelet Isolation

All experimental procedures involving collection of human and mouse blood were approved by the Ethic Committee of Xuzhou Medical University. Informed consent was obtained from all participants. Platelets were prepared from human and mouse blood as described previously (Qiao et al., 2017a; Qiao et al., 2018b). For human platelets, venous blood was collected into a tube anti-coagulated with trisodium citrate, glucose, and citric acid (ACD) and centrifuged for 20 min at 120 × *g* to obtain platelet-rich plasma (PRP) which was then centrifuged at 1,350 × *g* for 15 min and washed three times in CGS buffer. The platelet pellets were resuspended in Tyrode’s buffer. Mouse platelets were isolated from ACD anti-coagulated blood, washed, and resuspended in Tyrode’s buffer.

### Matrine Treatment

Human platelets were incubated with different doses of matrine (0, 0.25, 0.5, and 1 mg/ml) at 37°C for 1 h followed by relevant analysis.

### Platelet Aggregation and ATP Release

Platelet aggregation was conducted in the presence of fibrinogen (0.5 mg/ml) as described previously (Luo et al., 2019; Wei G. et al., 2020). After matrine treatment, platelet aggregation induced by CRP (0.1 μg/ml) or thrombin (0.04 U/ml) was analyzed in a Lumi-Aggregometer Model 700 (Chrono-log Corporation, Havertown, PA, United States) at 37°C with stirring (1,000 rpm). Platelet aggregation was presented as a percentage of maximum platelet aggregation. ATP release was monitored in parallel with platelet aggregation after addition of luciferin/luciferase reagent (Chrono-log Corporation) according to the manufacturer’s instructions and quantified relative to the vehicle (0 mg/ml matrine) treatment.

### Platelet α-granule Secretion and αIIbβ3 Activation

Platelet α-granule secretion was assessed via measuring P-selectin expression and integrin αIIbβ3 activation was evaluated via detecting the activation-dependent binding of PAC-1 to platelet αIIbβ3 by flow cytometry as described previously (Luo et al., 2019; Wei G. et al., 2020). Briefly, after matrine treatment, platelets were stimulated with CRP (0.1 or 2 μg/ml) or thrombin (0.04 or 0.5 U/ml) in the presence of PE-conjugated anti-P-selectin antibody or FITC-conjugated PAC-1 antibody followed by measuring P-selectin expression or PAC-1 binding by flow cytometry using FITC-conjugated mouse anti-human CD41a to set the platelet gate in the Forward Scatter and Side Scatter.

### Platelet Receptors Expression

After matrine treatment, FITC-conjugated mouse anti-human CD41a antibody (α_IIb_), FITC-conjugated anti-CD42b antibody (GPIbα), or anti-human GPVI antibody (detected by FITC-conjugated goat anti-mouse IgG) were added and incubated for 1 h followed by measuring the expression of platelet receptors by flow cytometry.

### Platelet Spreading

Platelets were placed on glass coverslips which were pre-coated with fibrinogen (10 μg/ml) or collagen (10 μg/ml) at 37°C for 90 min. After washing with PBS, platelets were fixed, permeabilized, stained with Alexa Fluor-546-labelled phalloidin and platelet spreading was observed under a fluorescence microscopy (Nikon-80i) using an x 100 oil objective. The surface covered area was quantified using ImageJ software.

### Clot Retraction

Thrombin-mediated clot retraction was initiated after addition of thrombin (1 U/ml) to washed platelets in the presence of 2 mM Ca^2+^ and 0.5 mg/ml fibrinogen as described previously (Luo et al., 2019; Wei G. et al., 2020).

### Tail Bleeding Assay

Mice received intraperitoneal injection of matrine (10 mg/kg). After 30 min, tail bleeding time was evaluated as described previously (Luo et al., 2019; Wei G. et al., 2020).

### FeCl_3_-Induced Arterial Thrombosis

After matrine (1 mg/ml) or vehicle treatment at 37°C for 1 h, mouse platelets (1 × 10^8^/ml) were labelled with calcein and infused into matrine-treated mice or wild-type mice respectively via tail vein injection. After 30 min, mesenteric arterioles damage was induced by 10% (w/v) FeCl_3_ and thrombus formation was monitored under a fluorescence microscopy (Olympus BX53).

### Deep Vein Thrombosis

Deep vein thrombosis model was established through ligation of the inferior vena cava (IVC) with a 2–0 nonabsorbable suture as described previously (Wei G. et al., 2020; Wang et al., 2020). After 24 h of ligation, thrombi were excised to measure the weight and length.

### Coagulation Factor Measurement

Plasma was extracted from matrine or vehicle-treated mice and the level of factor VIII and IX and prothrombin time and activated partial thromboplastin time was detected on an automated coagulation analyzer (Sysmex CS-5100).

### Measurement of ROS Production

After treatment with CRP (5 μg/ml) or thrombin (1 U/ml), platelet intracellular ROS generation was measured using 2′,7′-dichlorofluorescein (H2DCF-DA) as described previously (Wei et al., 2019; Wang et al., 2020).

### *In vitro* Thrombus Formation Under Arterial Flow Conditions

Human blood was labelled with mepacrine (100 μM) and perfused through fibrillar collagen (100 μg/ml)-precoated Bioflux plates in a microfluidic whole-blood perfusion assay (Bioflux-200 system) at a shear force of 40 dynes/cm^2^ for 5 min. Thrombus formation was dynamically monitored under an inverted fluorescence microscopy (Olympus IX53). The platelet-covered area was quantified using Bioflux software (Fluxion).

### Western Blotting

Human platelets were treated with CRP (5 μg/ml) or thrombin (1 U/ml) in the presence of different doses of matrine or vehicle for 5 min followed by measuring AKT (anti-Thr308 and anti-Ser473, Cell Signaling Technology; pan-AKT, Affinity Biosciences), ERK1/2 (anti-Thr202/Tyr204 and pan-ERK1/2, Cell Signaling Technology), p38 MAPK (anti-Thr180/Tyr182, Cell Signaling Technology), VASP (anti-Ser157, Affinity Biosciences; anti-Ser239 and pan-VASP, Cell Signaling Technology) by SDS-PAGE/western blot. The density of protein band was quantified using Image J software and the phosphorylation level was shown as a ratio to the total level.

For some experiments, the levels of c-Src (anti-Tyr-416, Cell Signaling Technology; pan-c Src, Proteintech) was measured in platelets after spreading on fibrinogen or bovine serum albumin (as a control), or under the condition of clot retraction (platelets were stimulated with thrombin (1 U/ml) in the presence of 2 mM Ca^2+^ and 0.5 mg/ml fibrinogen).

### Detection of cAMP and cGMP Level

The cAMP and cGMP level was measured by ELISA kit (Catalog number: 80203 and 80103) (NewEast Biosciences) according to the kit instructions.

### Statistical Analysis

Data are represented as mean ± standard error (SE). One-way ANOVA was performed for the comparison of difference among different groups. Two-way ANOVA with Bonferroni post-tests was performed for comparison among multiple groups over time. *p* < 0.05 indicates a statistical significance.

## Results

### Matrine Inhibits Platelet Aggregation and Dense-Granule Release

**FIGURE 1:**
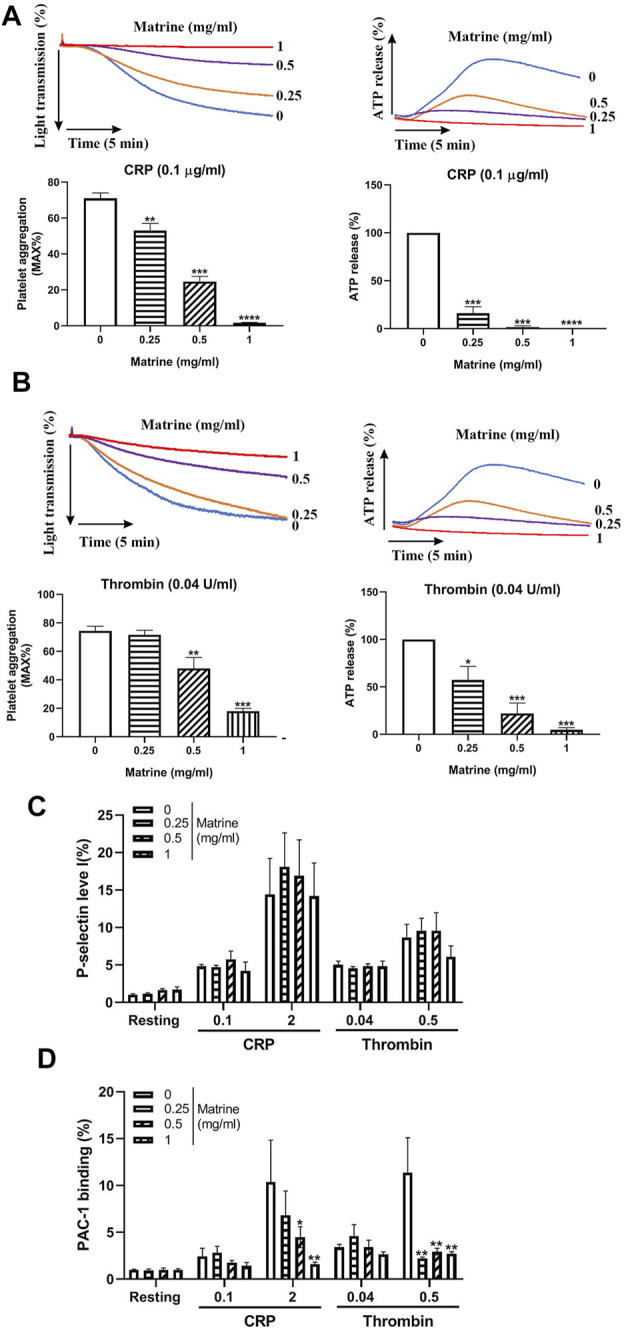
Platelet aggregation and granules release. Washed human platelets were treated with different doses of matrine (0, 0.25, 0.5, and 1 mg/ml) at 37°C for 1 h to measure platelet aggregation induced by CRP (0.1 μg/ml) **(A)** or thrombin (0.04 U/ml) **(B)** in a Lumi-Aggregometer. At the same time, ATP release was monitored simultaneously using luciferin/luciferase reagent and presented as a relative to 0 mg/ml matrine which was defined as 100%. After matrine treatment, platelet P-selectin expression (α-granule) **(C)** and integrin αIIbβ3 activation (PAC-1 antibody binding) **(D)** was measured after collagen or thrombin stimulation using PE-conjugated anti-P-selectin antibody or FITC-conjugated PAC-1 antibody by flow cytometry. Data were presented as mean ± SE (n = 3–5) and analyzed by one-way ANOVA. Compared to 0, ^*^ *p* < 0.05; **p < 0.01; ***p < 0.001.

Platelet aggregation is a critical process for platelet function. To evaluate whether matrine affects platelet aggregation, we treated human platelets with different doses of matrine (0, 0.25, 0.5, and 1 mg/ml) and then measured platelet aggregation in response to CRP or thrombin. As shown in Figure 1A, in comparison to vehicle treatment (0 mg/ml), matrine significantly inhibited platelet aggregation stimulated by CRP (0.1 μg/ml), or thrombin (0.04 U/ml) in a dose-dependent manner. Since thrombin might induce fibrinogen conversion to fibrin which might affect platelet agglutination, we also evaluated matrine’s effect on fibrin formation in the absence of platelets using human platelet-deficient plasma and found that matrine did not affect fibrin formation induced by thrombin as shown by no difference of thrombin time after thrombin stimulation in the presence of difference doses of matrine (Supplementary Figure S1). To further assess whether platelet dense-granule secretion is influenced by matrine, we simultaneously monitored ATP release (an indicator of platelet dense-granule secretion) during platelet aggregation induced by CRP or thrombin and found that matrine significantly decreased ATP release from CRP- or thrombin-stimulated platelets in a dose-dependent manner (Figure 1B). However, surprisingly, platelet α-granule secretion was not affected by matrine treatment after CRP or thrombin stimulation even at a higher dose as shown by no significant changes of P-selectin expression (an indicator of platelet α-granule secretion) (Figure 1C). Consistent with reduced platelet aggregation, matrine also significantly decreased integrin αIIbβ3 activation as demonstrated by the decrease of PAC-1 binding to platelet (Figure 1D). Taken together, this data show that matrine inhibits platelet aggregation, dense-granule release, and αIIbβ3 activation without affecting α-granule secretion.

### No Significant Changes of Platelet Receptors Expression After Matrine Treatment

**FIGURE 2:**
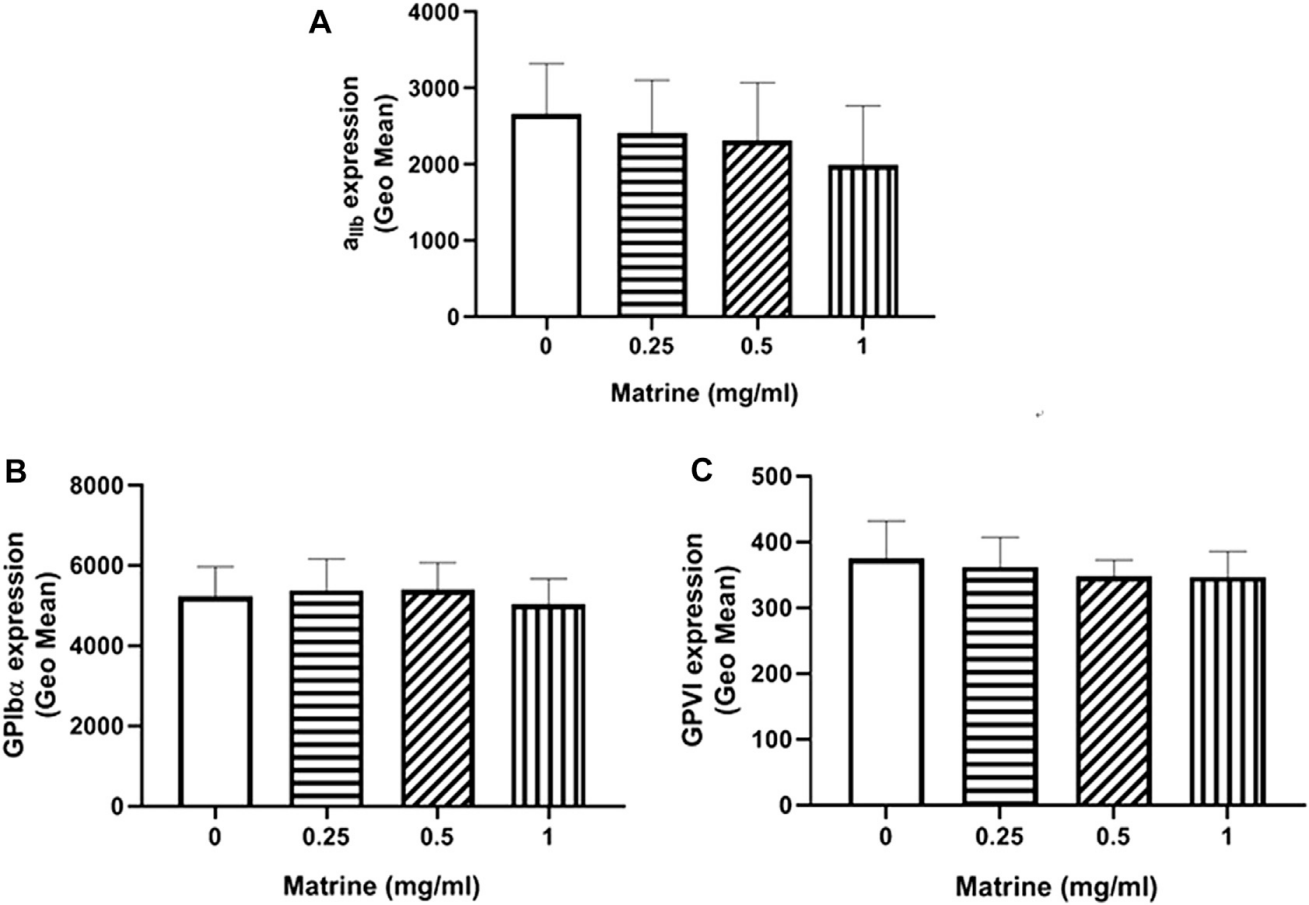
Surface expression level of platelet membrane receptors. After matrine treatment, the expression of platelet receptors α_IIb_β_3_ **(A)**, GPIbα **(B)** and GPVI **(C)** was detected by flow cytometry. Data were presented as mean ± SE (*n* = 6–7) and analyzed by one-way ANOVA.

Platelet membrane receptors GPIbα, GPVI, and α_IIb_β_3_ have been shown to play critical roles in the regulation of platelet aggregation via engagement of their respective ligand, such as von Willebrand factor (GPIbα), collagen (GPVI), and fibrinogen (α_IIb_β_3_) (Bennett, 2005; Gardiner and Andrews, 2014). Considering the impaired platelet aggregation induced by matrine, we measured the surface expression of these platelet receptors after matrine treatment by flow cytometry. As seen in Figure 2, matrine treatment did not alter the expression of GPIbα (Figure 2A), GPVI (Figure 2B), and α_IIb_β_3_ (Figure 2C) even at a higher dose as demonstrated by no significant change of the expression of these platelet receptors compared with vehicle (0 mg/ml matrine) treatment.

### Impaired Platelet Spreading and Clot Retraction After Matrine Treatment

**FIGURE 3:**
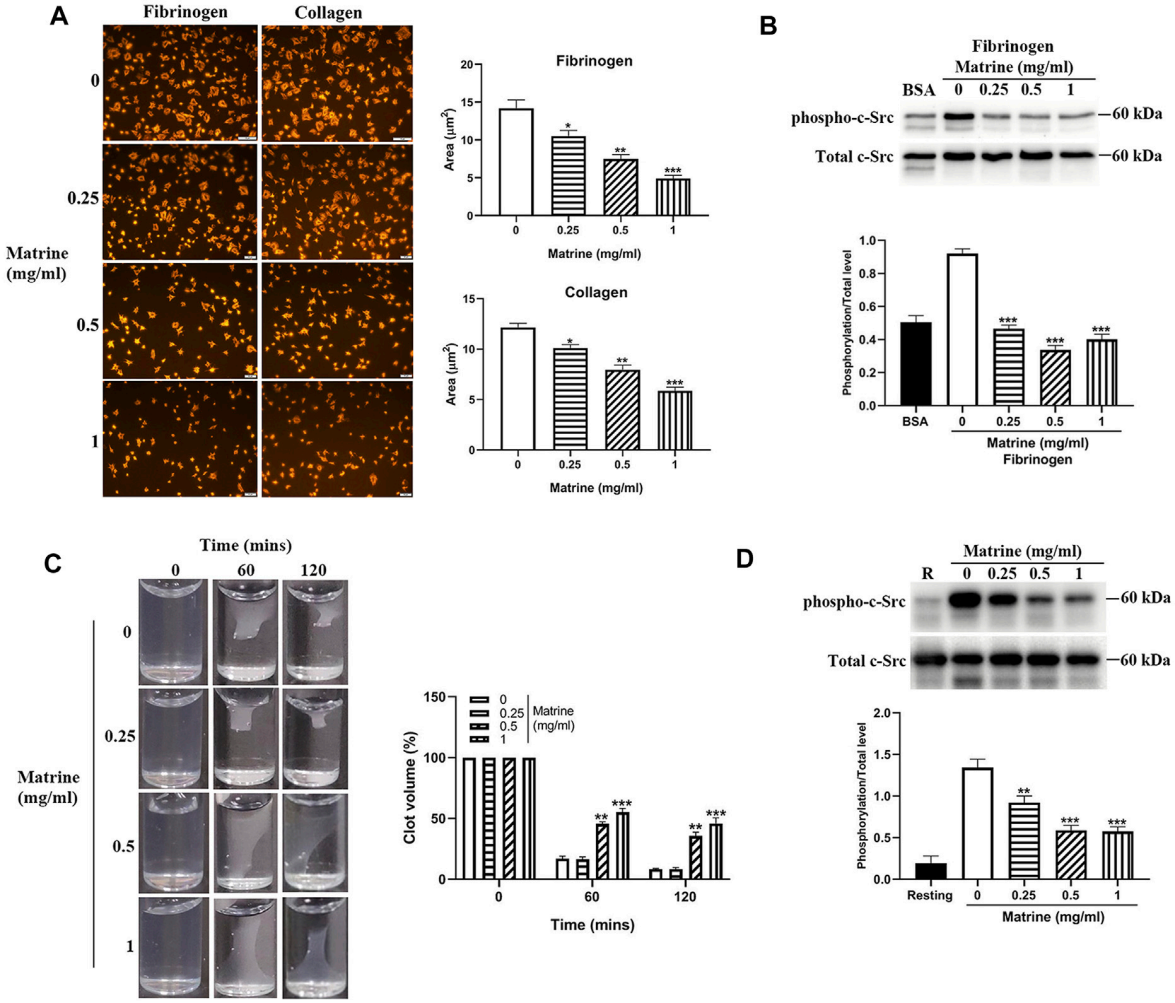
Platelet spreading and clot retraction. After matrine treatment, platelets were placed on fibrinogen or collagen coated glass coverslips and allowed to spread at 37°C for 90 min followed by staining with Alexa Fluor-546-labelled phalloidin **(A)** (mean ± SE, *n* = 3) or measurement of c-Src phosphorylation by western blot (mean ± SD, *n* = 3) **(B)**. Clot retraction was initiated in matrine-treated platelets in the presence of 2 mM Ca^2+^ and 0.5 mg/ml fibrinogen after addition of thrombin (1 U/ml). Images were captured every 30 min (mean ± SD, *n* = 3) **(C)**. Under clot retraction condition, the phosphorylation level of c-Src was measured by western blot and represented as a ratio relative to the total level (mean ± SD, *n* = 3) **(D)**. BSA: bovine serum albumin. Compared with 0, ^*^ *p* < 0.05; **p < 0.01; ***p < 0.001.

Ligands binding to αIIbβ3 not only mediates platelet aggregation, but also activates αIIbβ3 outside-in signaling which plays critical roles in the regulation of platelet spreading, clot retraction and thrombus stabilization. To further assess whether matrine plays a role in platelet spreading, we placed matrine-treated platelets on immobilized fibrinogen or collagen to allow them to spread and found significantly impaired platelet spreading on fibrinogen or collagen in a dose-dependent manner (Figure 3A). As activation of αIIbβ3 outside-in signaling causes the phosphorylation of c-Src, Syk, and PLCγ2, which regulates platelet spreading and clot retraction (Clark et al., 1994; Suzuki-Inoue et al., 2007), we then measured the phosphorylation status of c-Src in platelets after spreading on fibrinogen and found significantly reduced c-Src phosphorylation in matrine-treated platelets after spreading on fibrinogen (Figure 3B). Meanwhile, we also investigated matrine’s effect on clot retraction, a process regulated by α_IIb_β_3_ outside-in signaling (Durrant et al., 2017). In accordance with impaired platelet spreading, thrombin-mediated clot retraction in matrine-treated platelets was also significantly decreased as shown by the significantly increased clot volume of matrine-treated platelets compared with vehicle-treated platelets (Figure 3C). Consistently, matrine-treated platelets also presented a significantly reduced phosphorylation level of c-Src (Figure 3D) after thrombin stimulation under the condition of clot retraction.

### Matrine Impairs Hemostasis and Arterial Thrombus Formation

**FIGURE 4:**
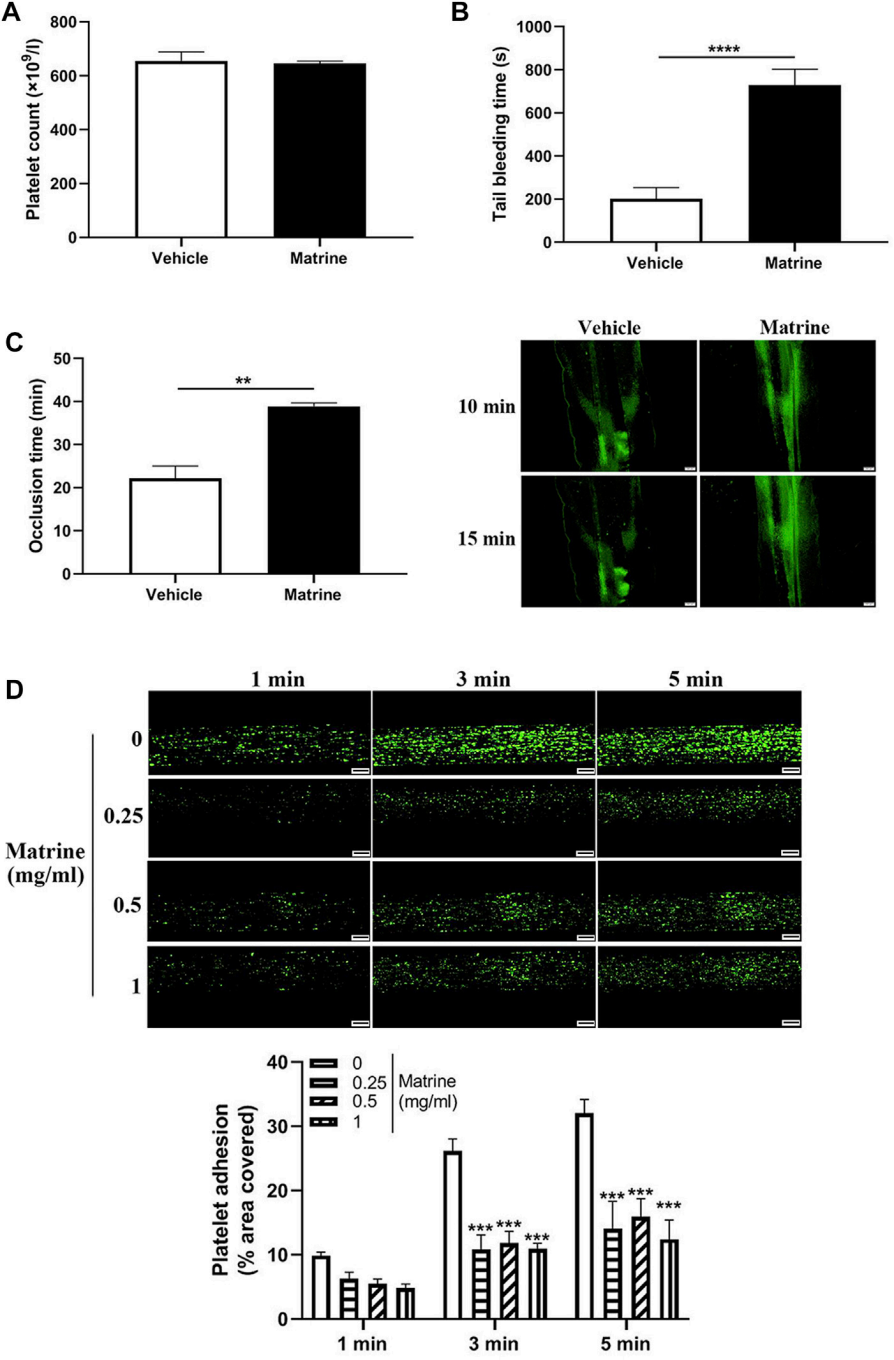
Effect of matrine on hemostasis and arterial thrombosis in mice**.** Mice received intraperitoneal injection of matrine (10 mg/kg) followed by analysis of platelet count **(A)**, tail bleeding time **(B)**, and arterial thrombus formation induced by FeCl_3_ which was monitored by a fluorescence microscopy (Olympus BX53) (mean, *n* = 6) **(C)**. Human blood labelled with mepacrine (100 μM) was perfused through Bioflux plates in a microfluidic whole-blood perfusion assay followed by monitoring the thrombus formation under a fluorescence microscopy. The platelet-covered area was quantified using Bioflux software (Fluxion) **(D)**. Scale bar = 100 μm. Data were presented as mean ± SE (*n* = 6–9). **p < 0.01 and ****p < 0.0001. Compared with 0, ***p < 0.001.

Given the inhibitory role of matrine in platelet function *in vitro*, we then investigated whether it affects *in vivo* platelet function through intraperitoneal injection of matrine into mice. As seen in Figure 4A, matrine administration into mice did not change the number of circulating platelets. However, matrine-administrated mice presented significantly prolonged the tail bleeding time compared with vehicle-treated mice (*p* < 0.0001) (Figure 4B). In addition, the arterial thrombus formation triggered by FeCl_3_ was also significantly delayed in matrine-treated mice infused with matrine-treated platelets compared with that in mice infused with vehicle-treated platelets (16.50 ± 1.36 min) (*p* < 0.01) (Figure 4C). Furthermore, we also assessed the effect of matrine on thrombus formation *in vitro* under arterial flow conditions using a microfluidic whole-blood perfusion system with collagen-coated BioFlux plates and found that the thrombus formation was significantly impaired after matrine treatment (Figure 4D). Taken together, these data indicate that matrine inhibits platelet hemostatic function and arterial thrombosis.

### Matrine Inhibits Venous Thrombus Formation *in Vivo*

**FIGURE 5:**
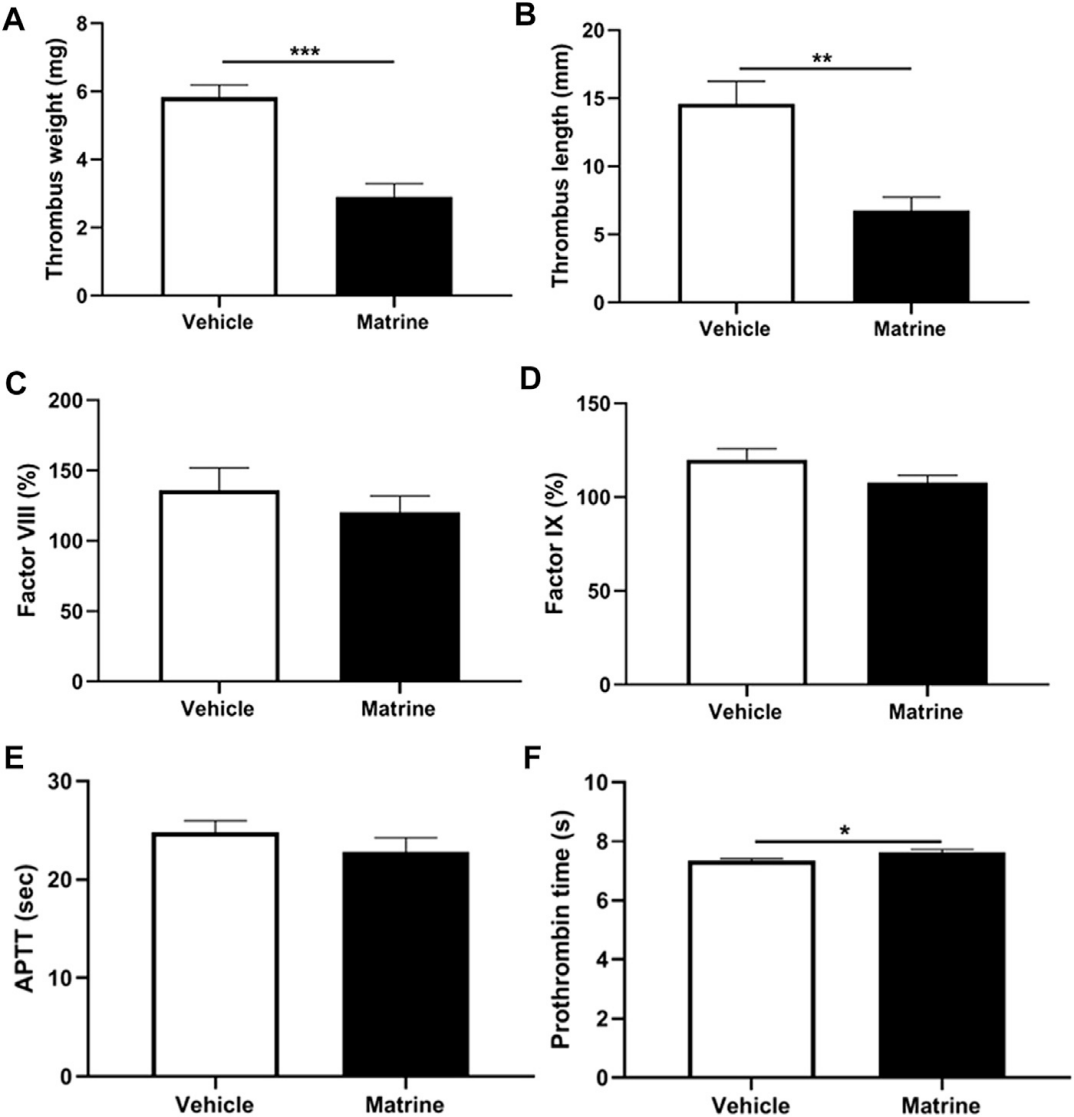
Deep vein thrombus formation and coagulation analysis. After intraperitoneal injection of matrine (10 mg/kg) or vehicle, mice underwent ligation of inferior vena cava (IVC) to induce venous thrombus formation. After 24 h, the IVC samples were isolated to measure thrombus weight **(A)** and length **(B)**. Meanwhile, peripheral blood was obtained from matrine or vehicle treated mice to detect the coagulation factor FVIII **(C)**, FIX **(D)**, activated partial thromboplastin time (APTT) **(E)** and prothrombin time **(F)**. Data were presented as mean ± SE (*n* = 4–6). **p < 0.01; ***p < 0.001.

Except arterial thrombosis, platelets have also been demonstrated to play a role in venous thrombus formation in recent years (Montoro-García et al., 2016). To further analyze whether matrine exerts an effect on venous thrombosis, we established a deep vein thrombosis model through IVC ligation and found that matrine treatment significantly inhibited venous thrombus formation as demonstrated by a significantly reduced thrombus weight (*p* < 0.001) (Figure 5A) and shortened thrombus length (*p* < 0.01) compared to vehicle treatment (Figure 5B). To assess whether coagulation participates in the decreased venous thrombus formation induced by matrine, we detected the level of coagulation factor VIII (Figure 5C), IX (Figure 5D), prothrombin time (Figure 5E), and activated partial thromboplastin time (Figure 5F) and found no significant difference of these coagulation factors and prothrombin time except a slight increase of activated partial thromboplastin time in mice administrated with matrine compared to vehicle. Taken together, these data show that matrine inhibits venous thrombus formation.

### Matrine Inhibits ROS Generation From Platelets After Stimulation

**FIGURE 6:**
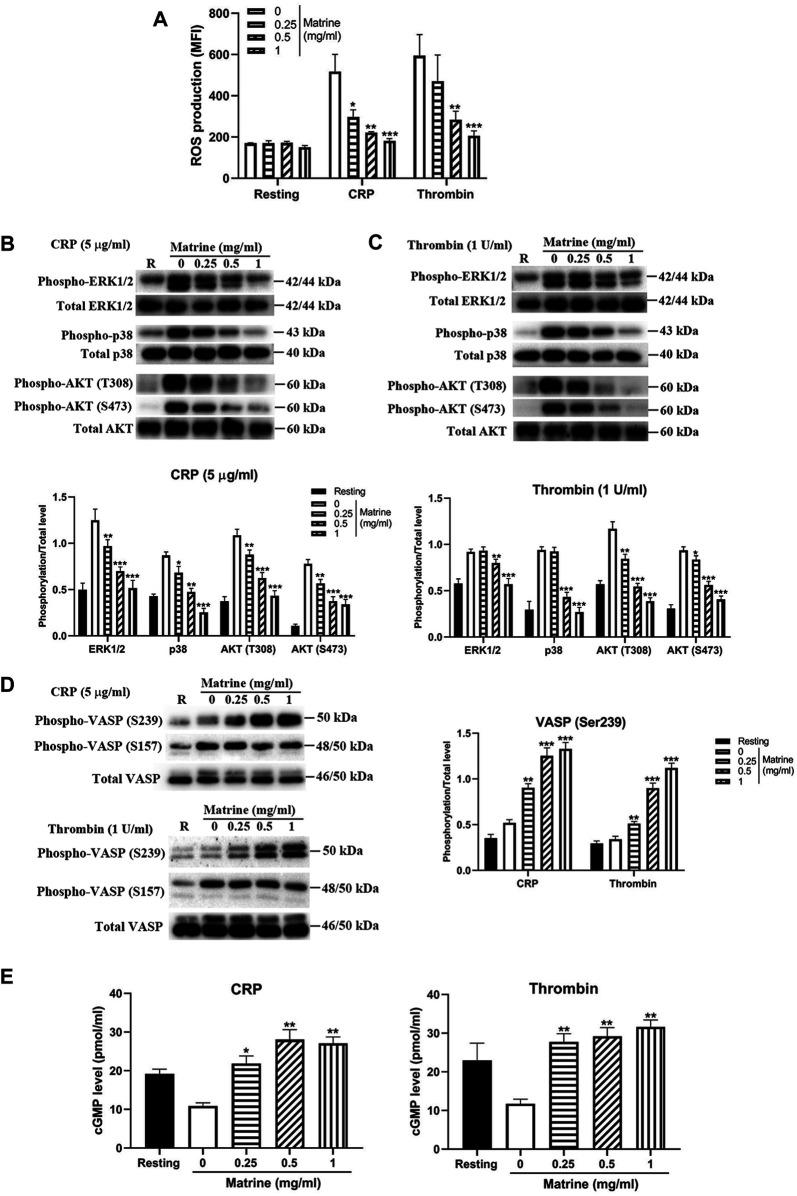
ROS generation, phosphorylation of ERK1/2, p38, AKT and VASP and cGMP level. After matrine treatment, platelet ROS generation was measured using H2DCF-DA by flow cytometry **(A)**. Matrine-treated platelets were stimulated with 5 μg/ml CRP or 1 U/ml thrombin for 5 min to measure the phosphorylation level of ERK1/2, p38, AKT **(B and C)** or VASP **(D)** by western blot (mean ± SD, *n* = 3), cGMP level by ELISA (mean ± SE, *n* = 3) **(E)**. Compared with 0, ^*^ *p* < 0.05; **p < 0.01; ***p < 0.001.

Since several studies have shown that matrine has antioxidant property via inhibition of the oxidative stress under pathological conditions, we then evaluated whether matrine affects platelet ROS production and found that matrine treatment significantly decreased ROS generation from platelets after stimulation with CRP (5 μg/ml) or thrombin (1 U/ml) compared with vehicle treatment (Figure 6A), indicating that matrine also possesses antioxidant effect on platelets. As mitogen-activated protein kinases (MAPKs), consisting of extracellular signal-related kinases 1/2 (ERK1/2), c-jun NH2-terminal kinases (JNK), and p38 MAPK, are sensitive to oxidative stress (Son et al., 2011), we then measured whether matrine treatment alters the phosphorylation of ERK1/2 and p38. As seen in Figures 6B,C, matrine-treated platelets presented significantly reduced phosphorylation level of ERK1/2 and p38 after CRP (Figure 6B) or thrombin (Figure 6C) stimulation. Meanwhile, we also found that matrine impaired AKT signaling in activated platelets as demonstrated by the reduced phosphorylation level of AKT (T308 and S473) in matrine-treated platelets after stimulation with CRP or thrombin. We next evaluated matrine’s effect on PKA/PKG activation via measuring the phosphorylation of VASP (Ser157/239) as the Ser157 site is the major PKA phosphorylation site and Ser239 is the major PKG phosphorylation site (Doppler and Storz, 2013). As seen in Figure 6D, matrine treatment significantly promoted the phosphorylation of VASP (Ser239) in CRP or thrombin-treated platelets without altering Ser157 phosphorylation, consistent with the inhibitory role of VASP phosphorylation in platelet function (Aszodi et al., 1999), suggesting that matrine exerts anti-platelet effect possibly through activation of PKG signaling. In accordance with this, matrine also significantly enhanced cGMP level (Figure 6E) without affecting cAMP level (Supplementary Figure S2) in CRP or thrombin-stimulated platelets.

## Discussion

Matrine is the main bioactive compound in *Kushen* and has several pharmacological effects (You et al., 2020; Zhang et al., 2020), such as anti-cancer, anti-oxidant, anti-inflammatory properties. With these pharmacological properties, matrine has been widely used in the treatment of several diseases (You et al., 2020; Zhang et al., 2020), such as cancers, myocardial ischemia, rheumatoid arthritis, and Alzheimer’s disease. Considering the wide broad spectrum activities of matrine, whether it affects platelet function and thrombus formation remains poorly understood. In the present study, we evaluated the effect of matrine on platelet function and demonstrated that matrine inhibits platelet function, *in vivo* hemostasis, arterial thrombosis, and venous thrombosis, indicating that matrine might be used as a novel antiplatelet agent for the treatment of thrombotic or cardiovascular diseases.

The pharmacological effects of matrine on cancer cells has been widely investigated (Liu et al., 2010; Zhang et al., 2011; Liu et al., 2014b). In addition, matrine participates in the reversion of multidrug resistance through regulating the expression of several proteins, such as P-glycoprotein (P-gp), multidrug resistance-related protein (MRP) (Chai et al., 2010; Zhou et al., 2018). Moreover, matrine has anti-inflammatory effects via regulation of the expression of several inflammatory cytokines (such as TNF-α, IL-1) or signaling (such as NF-κB) or the inflammatory mediators (nitric oxide and matrix metalloproteinases) (You et al., 2020). In this study, we showed that matrine also exerts antiplatelet effect as demonstrated by a significant decrease of platelet aggregation, dense granule release, platelet spreading, and clot retraction after matrine treatment compared to vehicle treatment. Furthermore, matrine also possess *in vivo* anti-thrombotic properties since matrine-treated mice presented significantly prolonged tail bleeding time and delayed arterial and venous thrombus formation, indicating that matrine might also be used a novel potential anti-thrombotic drug. However, our study found that matrine only affects platelet dense granule secretion but not alpha granule release as demonstrated by no difference of P-selection expression in the presence of difference doses of matrine after CRP or thrombin stimulation. This could be due to the different roles of dense granule release and alpha granule release in the regulation of platelet function (McNicol and Israels, 1999; Yadav and Storrie, 2017) as demonstrated by enhanced platelet response to low dose of agonists by ATP or ADP which is secreted from dense granules after stimulation (Farndale et al., 2004).

In response to vascular injury, adhesion of platelets to damaged vessel wall is the first step for platelets to repair the injured vessel. Platelet glycoprotein receptors, GPIbα and GPVI are primary platelet adhesive receptors to regulate platelet adhesion and function through recognition of exposed VWF and collagen respectively (Rivera et al., 2009). Engagement of platelet receptors will cause the transduction of intra-platelet signaling pathway and α_IIb_β_3_ activation, which binds to fibrinogen or VWF and mediates platelet aggregation and thrombus formation (Rivera et al., 2009). Considering the inhibition of platelet aggregation by matrine, the surface expression of these receptors after matrine treatment was measured. Surprisingly, we found that matrine treatment did not alter the expression profile of these platelet receptors, indicating that matrine inhibits platelet aggregation and function without affecting the expression of platelet surface receptors.

Reactive oxygen species (ROS) are natural by-products of aerobic metabolism and regulate multiple intracellular signaling pathways under physiological and pathological conditions (Finkel, 2011; Brown and Griendling, 2015). In recent years, ROS plays a role in the regulation of platelet function and thrombus formation (Qiao et al., 2017b) and antioxidants have been applied for the prevention and treatment of several thrombotic or cardiovascular diseases (Núñez-Córdoba and Martínez-González, 2011; Goszcz et al., 2015). Multiple studies demonstrated that matrine has antioxidant property through inhibition of the oxidative stress under pathological conditions. Matrine is capable to inhibit ROS production induced by advanced glycation end products (AGEs) in human aortic endothelial cells (HAEC) *in vitro* (Zhang et al., 2018). Meanwhile, matrine administration decreased malondialdehyde level and increased the level of superoxide dismutase, glutathione peroxidase, and total antioxidant capacity in MCAO-induced cerebral I/R injury mouse model (Zhao et al., 2015) and a d-galactose- (D-gal-) induced aging mouse model (Sun et al., 2018). Consistent with the antioxidant effect of matrine, our study showed that matrine treatment significantly inhibited ROS generation in platelets after stimulation with CRP or thrombin, indicating that matrine exerts antiplatelet effect possibly through regulation of intra-platelet ROS generation. Therefore, matrine-induced the decrease of ROS generation might contribute to the reduced phosphorylation of c-Src in platelets after spreading on fibrinogen or under clot retraction condition since Src protein tyrosine kinase activity can be regulated by redox (Giannoni et al., 2005; Sun and Kemble, 2009). Furthermore, we observed that the phosphorylation level of ERK1/2 (Thr202/Tyr204) and p38 MAPK (Thr180/Tyr182) was significantly reduced in matrine-treated platelets after stimulation, which was consistent with multiple studies showing that MAPK signaling pathways are sensitive to oxidative stress which is caused by the changes of ROS (Gaitanaki et al., 2003; Son et al., 2011). Moreover, we demonstrated that matrine also inhibited platelet AKT signaling transduction after stimulation, in accordance with previous studies demonstrating the inhibitory effect of matrine on PI3K/AKT signaling pathway (Niu et al., 2014; Liu et al., 2017; Wei J. et al., 2020). Furthermore, matrine treatment also significantly increased the phosphorylation of phosphorylation of VASP (Ser239) and the intracellular cGMP level, consistent with the inhibitory role of VASP phosphorylation and cGMP in platelet function (Aszodi et al., 1999). However, given that nicotinamide adenine dinucleotide phosphate (NADPH) oxidase (NOX) is the main source of ROS in activated platelets, followed by cyclooxygenase (COX), xanthine oxidase (XO), and mitochondrial respiration (Wachowicz et al., 2002), the exact molecular mechanism by how matrine inhibits platelet ROS generation, possibly via targeting NOX, COX, XO, or mitochondria requires further investigations.

In conclusion, matrine inhibits platelet function, hemostasis, arterial, and venous thrombus formation *in vivo*, which might be via direct or indirect inhibition of ROS generation, suggesting it may represent a novel inhibitor of platelet function and thrombus formation. However, caution should be paid to the potential bleeding risk in case of using matrine as an anti-thrombotic agent since matrine inhibits the hemostatic function of platelets in mice.

## Data Availability

The raw data supporting the conclusions of this article will be made available by the authors, without undue reservation.
