# Supplementary figures and images for: Matrine Impairs Platelet Function and Thrombosis and Inhibits ROS Production
Source: Front Pharmacol. 2021 Jul 22;12:717725. doi: 10.3389/fphar.2021.717725 (PMC8339414; doi:10.3389/fphar.2021.717725)

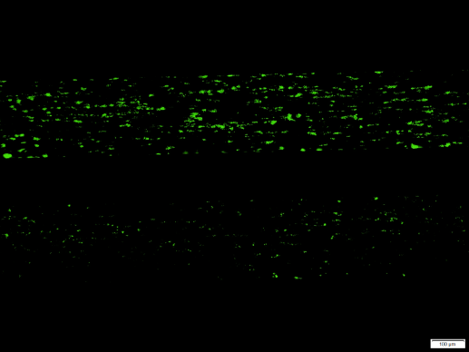

Supplement: Supplementary file 1 [file DataSheet1.ZIP › Raw data (3)/Raw data/Flow chamber original images/1 min-0-0.25.tif]

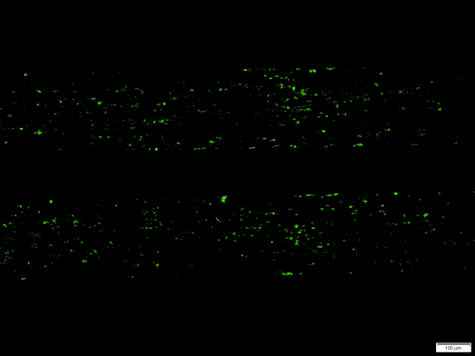

Supplement: Supplementary file 1 [file DataSheet1.ZIP › Raw data (3)/Raw data/Flow chamber original images/1 min-0.5-1.tif]

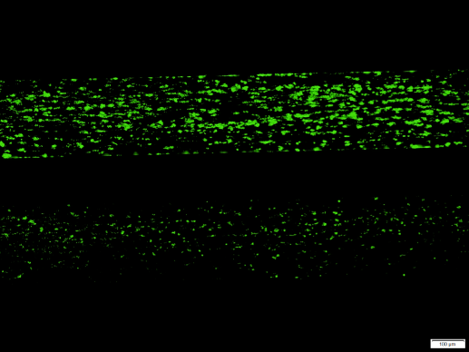

Supplement: Supplementary file 1 [file DataSheet1.ZIP › Raw data (3)/Raw data/Flow chamber original images/3 min-0-0.25.tif]

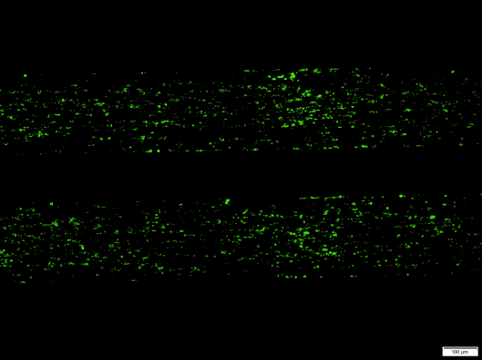

Supplement: Supplementary file 1 [file DataSheet1.ZIP › Raw data (3)/Raw data/Flow chamber original images/3 min-0.5-1.tif]

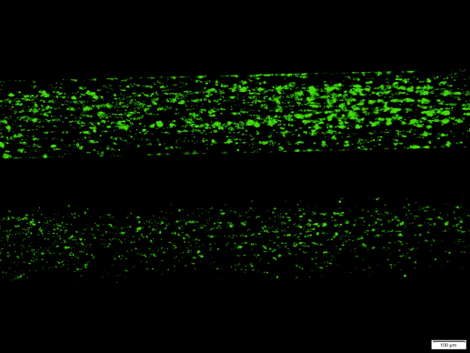

Supplement: Supplementary file 1 [file DataSheet1.ZIP › Raw data (3)/Raw data/Flow chamber original images/5 min-0-0.25.tif]

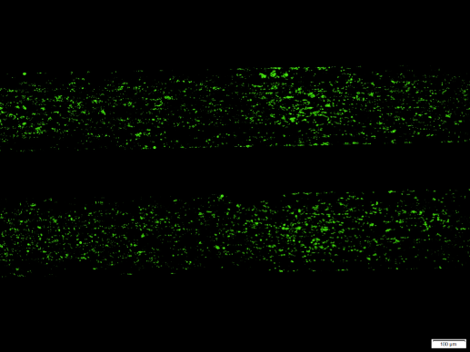

Supplement: Supplementary file 1 [file DataSheet1.ZIP › Raw data (3)/Raw data/Flow chamber original images/5 min-0.5-1.tif]

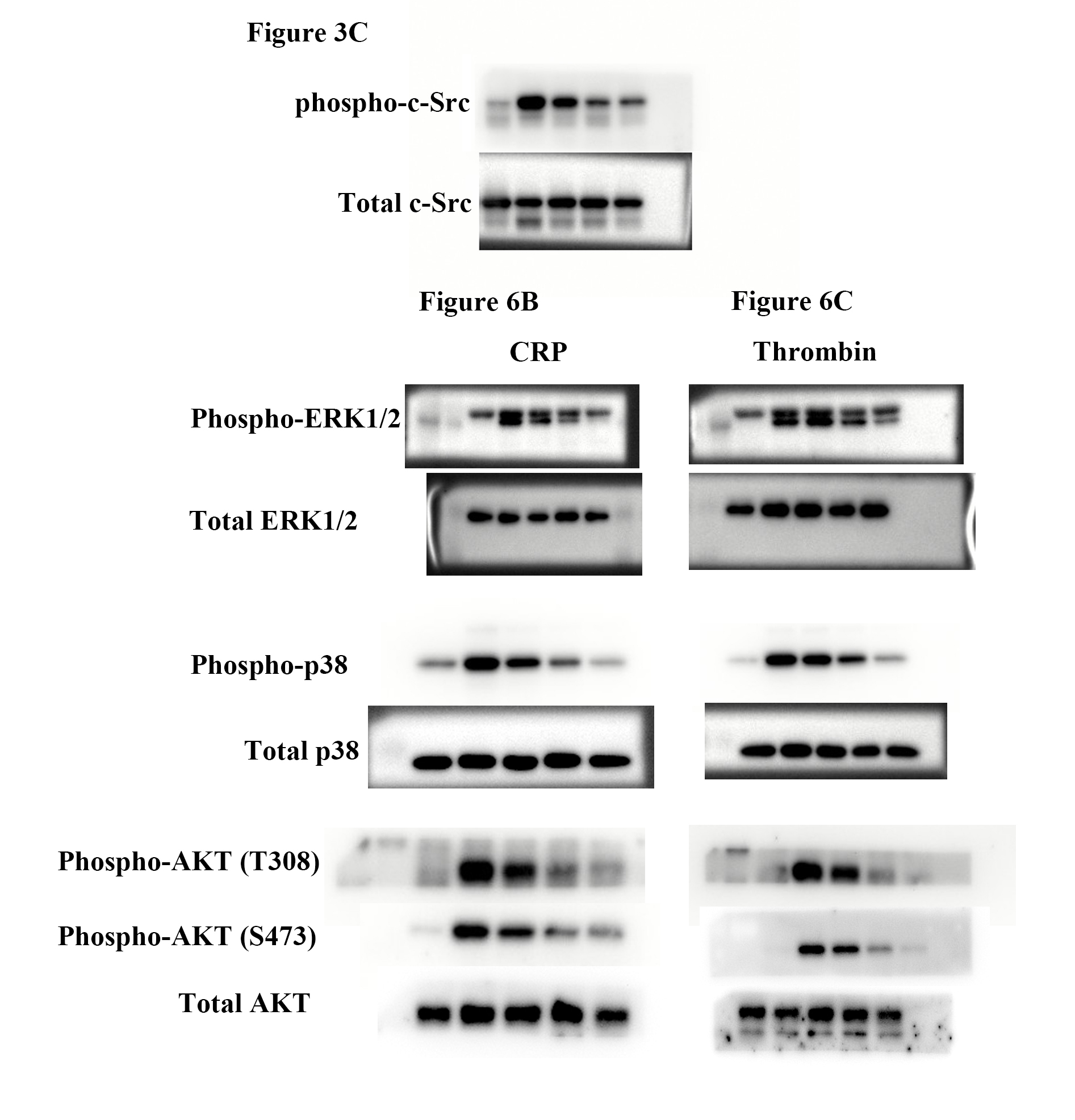

Supplement: Supplementary file 1 [file DataSheet1.ZIP › Raw data (3)/Raw data/Original gel images.jpg]

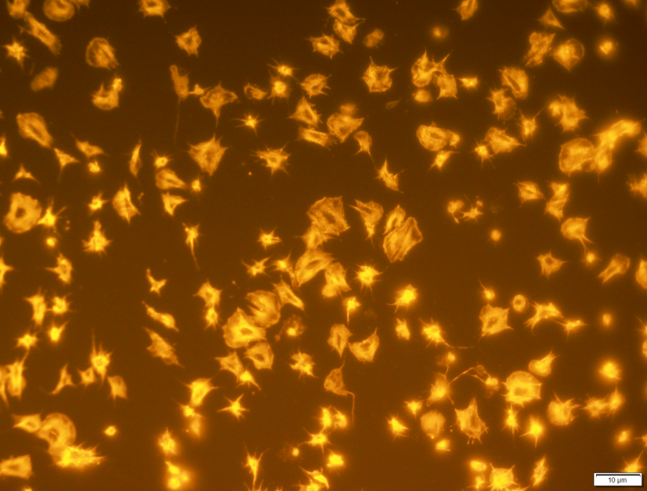

Supplement: Supplementary file 1 [file DataSheet1.ZIP › Raw data (3)/Raw data/Platelet spreading original images/COLL 0.25.tif]

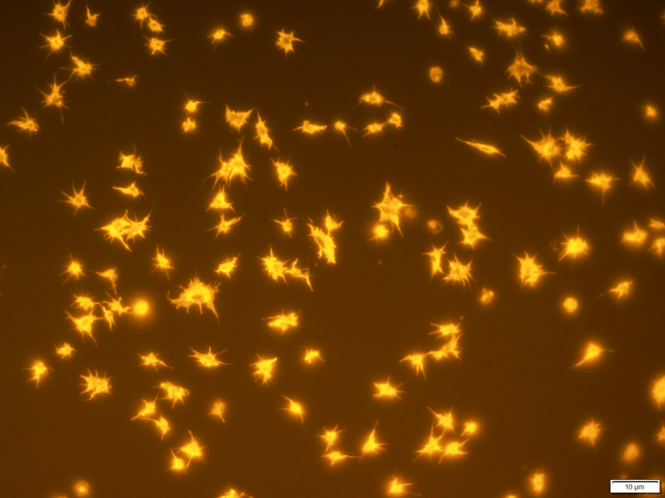

Supplement: Supplementary file 1 [file DataSheet1.ZIP › Raw data (3)/Raw data/Platelet spreading original images/COLL 0.5.tif]

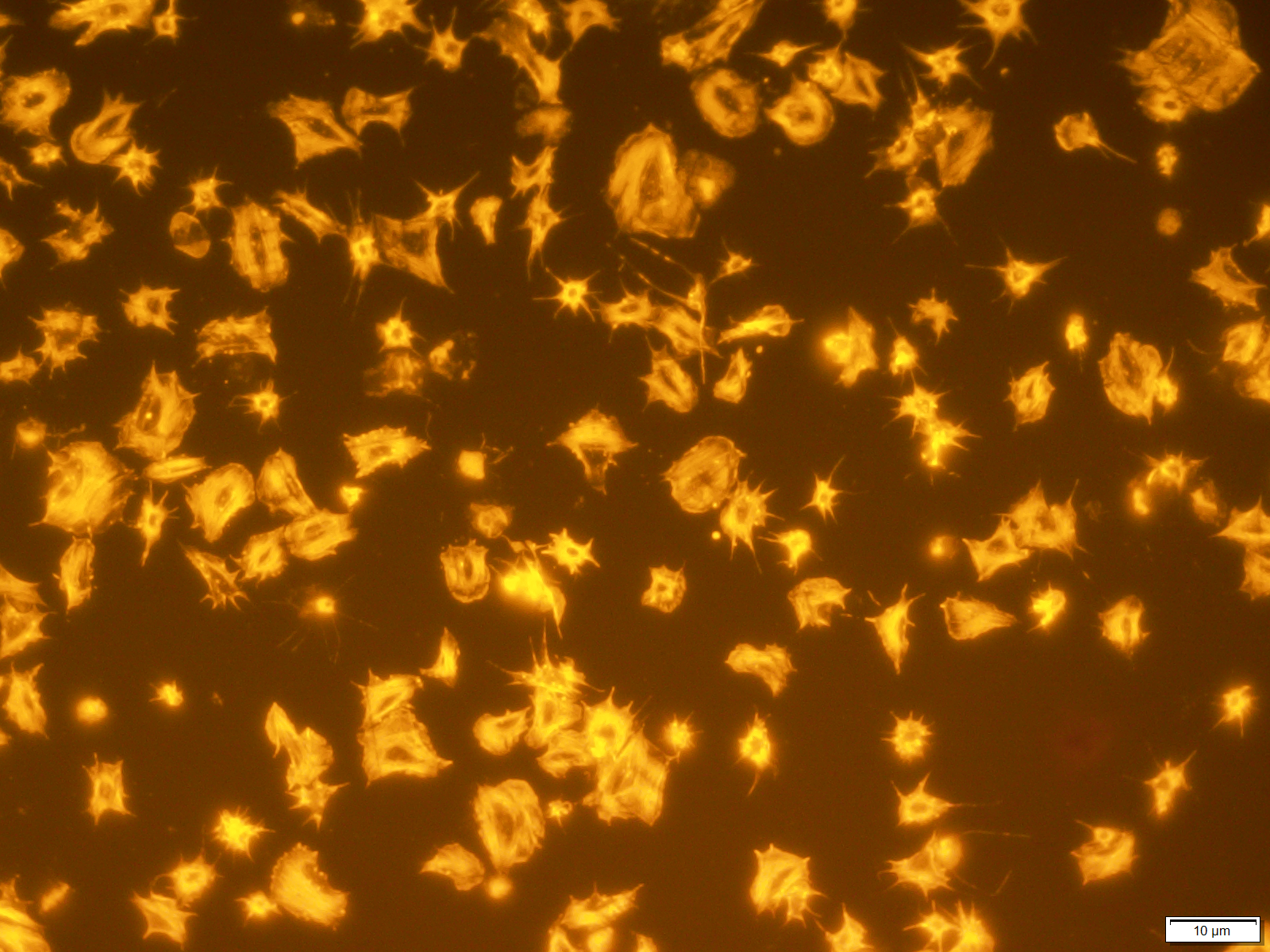

Supplement: Supplementary file 1 [file DataSheet1.ZIP › Raw data (3)/Raw data/Platelet spreading original images/COLL 0.tif]

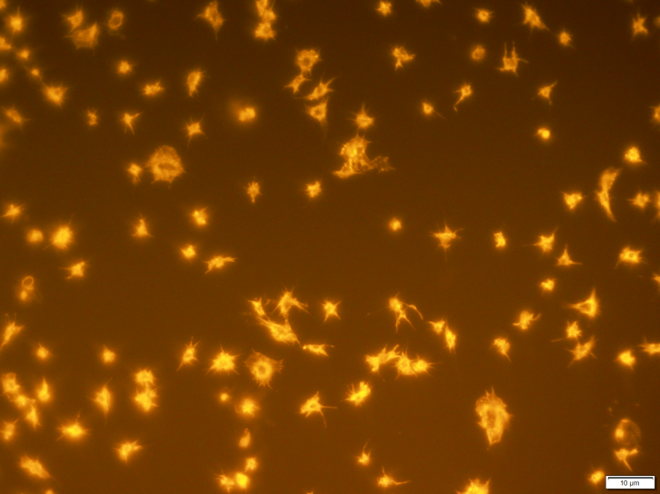

Supplement: Supplementary file 1 [file DataSheet1.ZIP › Raw data (3)/Raw data/Platelet spreading original images/COLL 1.tif]

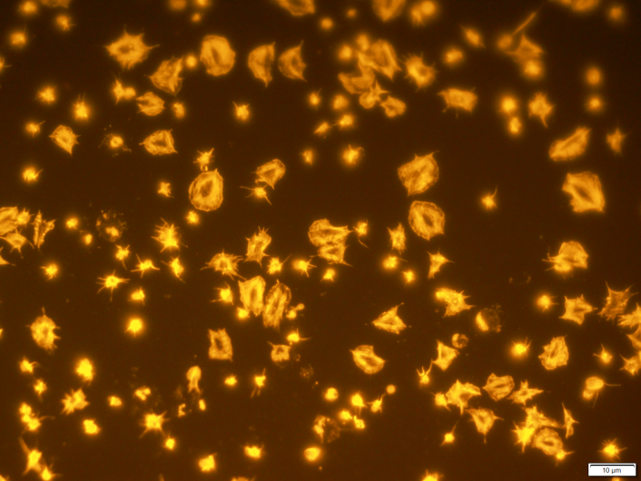

Supplement: Supplementary file 1 [file DataSheet1.ZIP › Raw data (3)/Raw data/Platelet spreading original images/FNG 0.25.tif]

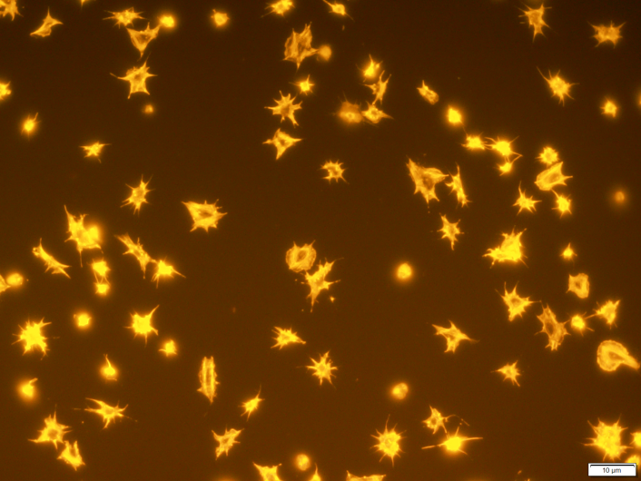

Supplement: Supplementary file 1 [file DataSheet1.ZIP › Raw data (3)/Raw data/Platelet spreading original images/FNG 0.5.tif]

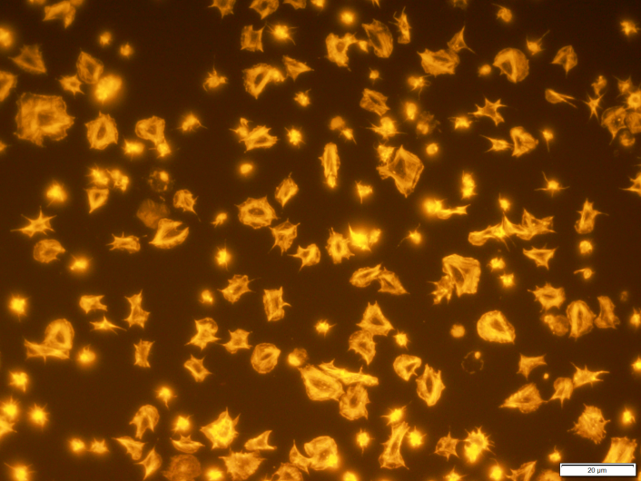

Supplement: Supplementary file 1 [file DataSheet1.ZIP › Raw data (3)/Raw data/Platelet spreading original images/FNG 0.tif]

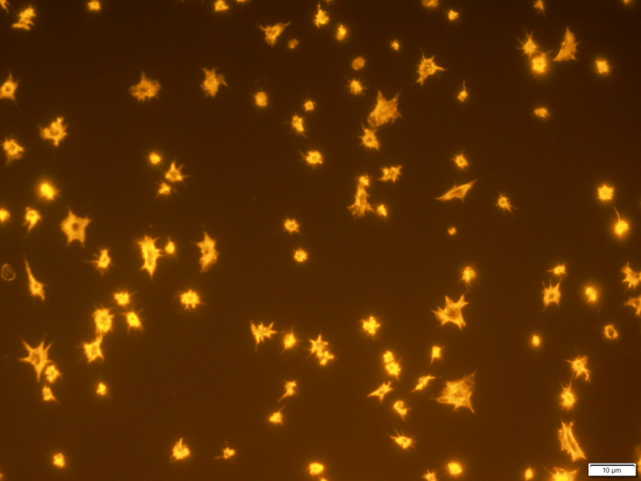

Supplement: Supplementary file 1 [file DataSheet1.ZIP › Raw data (3)/Raw data/Platelet spreading original images/FNG 1.tif]
