# Supplementary material for: Matrine Impairs Platelet Function and Thrombosis and Inhibits ROS Production
Source: Front Pharmacol. 2021 Jul 22;12:717725. doi: 10.3389/fphar.2021.717725 (PMC8339414; doi:10.3389/fphar.2021.717725)
Supplement: Supplementary file 2 [file DataSheet2.docx]

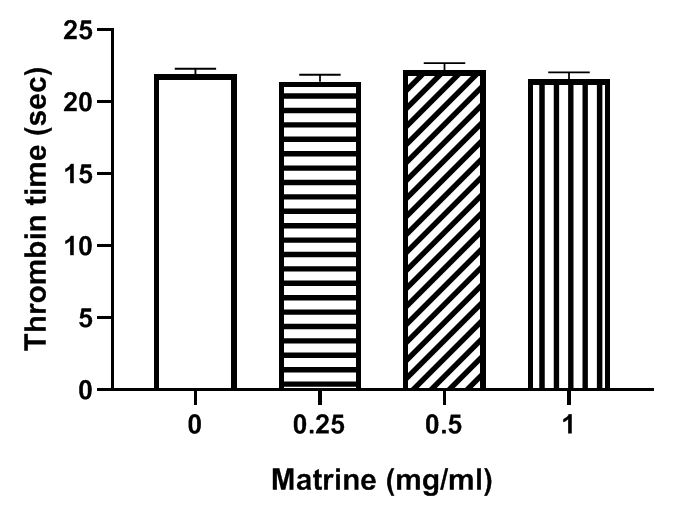


**Figure S1. Thrombin time in platelet-deficient plasma.** Platelet-deficient plasma was isolated from healthy individuals and stimulated with thrombin (0.04 U/ml) in the presence of different doses of matrine followed by measurement of thrombin time. Data were presented as mean ± SE (n=3).

**
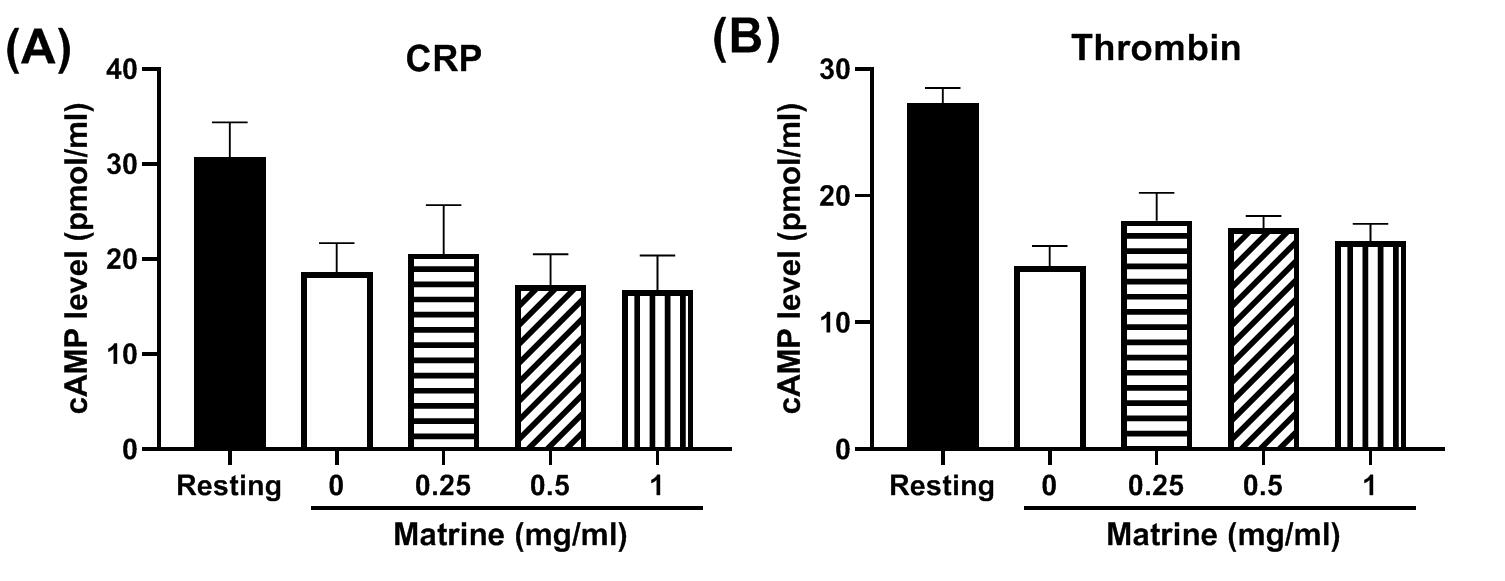
**

**Figure S2. cAMP level in platelets after matrine treatment.** After matrine treatment, platelets were stimulated with 5 μg/ml CRP (A) or 1 U/ml thrombin (B) for 5 min followed by measuring intracellular cAMP level by ELISA (mean ± SE, n = 3-5).
